# Trends in avoidable hospitalizations in a developed City in eastern China: 2015 to 2018

**DOI:** 10.1186/s12913-022-08275-w

**Published:** 2022-07-04

**Authors:** Siyuan Chen, Hongqiao Fu, Weiyan Jian

**Affiliations:** Department of Health Policy and Management, School of Public Health, Peking University, No. 38, Xueyuan Road, Haidian District, Beijing, 100191 China

**Keywords:** Avoidable hospitalization, Healthcare expenditure, Length of stay, Primary health care, Quality of care

## Abstract

**Objective:**

This study aimed to measure the avoidable hospitalization rate and the treatment cost per hospitalization in large cities of eastern China.

**Methods:**

In this study, the hospital discharge data of all inpatients in the city from 2015 to 2018 were collected. In accordance with the organization for Economic Cooperation and Development (OECD) definition of avoidable hospitalizations, five diseases were selected as the measurement objects, including hypertension, diabetes, asthma, chronic obstructive pulmonary disease (COPD), as well as congestive heart failure (CHF). We described the avoidable hospitalization rate, average cost and length of stay for avoidable hospitalization cases. Linear probability model and log-linear model were used to control the basic characteristics and disease severity of patients, and to measure the trend of the avoidable hospitalization rate and expenditure of avoidable hospitalizations.

**Results:**

From 2015 to 2018, the absolute number of avoidable hospitalizations in the city increased while fluctuating, which reached 125,372 in 2018. Among the five avoidable hospitalizations, the number of hospitalizations for diabetes increased continuously in the 4-year period. Congestive heart failure showed the most significant increase over the four years. Avoidable hospitalizations in the city have remained at a high level, while avoidable hospitalizations of hypertension and asthma fell to levels lower than those in 2015 in 2017 and 2018 after rising in 2016. The cost per hospitalization and length of stay per hospitalization decreased.

**Conclusions:**

Avoidable hospitalizations in the city remain at a high level, and more effective policies should be formulated to guide patients with avoidable hospitalizations, so as to more effectively exploit outpatient services and continuously improve the quality of primary health care services.

**Supplementary Information:**

The online version contains supplementary material available at 10.1186/s12913-022-08275-w.

## Introduction

It has been widely accepted that numerous hospitalizations can be avoided by timely and effective outpatient care. Avoidable hospitalizations can be avoided by timely and effective primary health care services [1, 2]. In general, avoidable hospitalizations are separated into three types [3, 4]: first, diseases of which the occurrence can be reduced, thus avoiding hospitalization by vaccination, which include infectious diseases (e.g., measles, pertussis and tetanus); second, acute diseases that can be appropriately controlled and treated using timely and effective out-of-hospital symptomatic treatments, which consist of gastroenteritis, urinary tract infections, as well as bacterial pneumonia; third, acute diseases that can be prevented by quality out-of-hospital services, as well as chronic diseases that aggravate or reduce the incidence of complications, which include diabetes, hypertension, asthma, etc. Several international organizations have formulated standards relating to avoidable hospitalization. To be specific, the standards formulated by the Institute for Health Services and Quality in the United States involve 19 avoidable hospitalization conditions [5], those developed by the Universal Health Service in the United Kingdom cover 19 avoidable hospitalization conditions [4], those formulated by the Canadian Institute for Health Information have only seven conditions [6], and those of the OECD contain only 6 diseases [7]. The above standards all contain the same six diseases, including hypertension, diabetes, asthma, chronic obstructive pulmonary disease (COPD), as well as congestive heart failure (CHF). All of the above conditions are chronic diseases without self-limiting disease progression and generally result in subsequent hospitalization if necessary out-of-hospital care is lacking beforehand.

Much evidence indicates that lack of access to primary health care services can result in avoidable hospitalizations, expose patients to risks in the hospital setting (e.g., infections and depression) and cause significant avoidable health costs to the NHS [8–11]. This significant correlation has led to avoidable hospitalization rates that are extensively used as a marker of primary health care effectiveness [11]. Over the past few years, avoidable hospitalizations have been increasingly employed to evaluate patients’ access to and quality of primary care [4, 9, 12].

One study [13] obtained age- and sex-standardized rates of avoidable hospitalizations in several European nations using a combined indicator of avoidable hospitalizations consisting of six chronic diseases. The above study found that the standardized rate of avoidable hospitalizations in 2009 was 93.7 per 10,000 inhabitants in Denmark, 56.6 per 10,000 inhabitants in the United Kingdom, and 34.8 per 10,000 inhabitants in Portugal. An Italian study [14] selected the total number of hospitalizations as the denominator of the avoidable hospitalization rate and obtained the avoidable hospitalization rate of Sicilian residents as 3.4% for chronic diseases, 2.5% for acute diseases, as well as 0.4% for vaccinations. Another study in Singapore [10] selected the total number of hospitalizations as the denominator of the avoidable hospitalization rate and selected five chronic avoidable hospitalizations, and it was reported that the avoidable hospitalization rate in Singapore reached 6.7% for 1991–1998. In other studies, the primary health care quality indicators published by the OECD were employed to identify avoidable hospitalizations for diabetes, and the avoidable hospitalization rate was estimated for those diagnosed with diabetes (i.e., the number of diabetes-related avoidable hospitalizations in a given year divided by the number of people diagnosed with diabetes in the same location and year). As reported by the study, the avoidable hospitalization rate per 1000 patients suffering from diabetes in 2012–14 in Japan was 23.3, 36.2 in Singapore and 42.5 in Hong Kong [15]. Several studies were conducted on individual avoidable hospitalizations; for instance, the avoidable hospitalization rate of uncomplicated hypertension was 3.7 per 1000 hypertensive patients in four Canadian provinces and territories [16].

In mainland China, studies on avoidable hospitalization have had a relatively late start and have been rarely conducted. Using national total health cost monitoring data, Pei-Pei Chai et al. performed a national-level measurement in 2019 by caliber of eight avoidable chronic diseases, including COPD, asthma, bronchiectasis, diabetes, hypertension, angina pectoris, heart failure, as well as rheumatic heart disease; as revealed by the results, the avoidable hospitalization rate was obtained as 9.53% [17]. Fei-Cheng Li [18] and Si-Yuan Chen [19] measured the avoidable hospitalization rate of diabetes and hypertension as 9.53 and 3.53%, respectively. Some other studies measured using information from only a few hospitals [20]. There have been rare analyses on trends in avoidable hospitalization rates and treatment costs, and the only analyses that have been conducted have used older data [15].

China’s hospitalization rate has been rising steadily, reaching 13.7% in 2018 [21], which has made it unaffordable for health insurance funds in the long run, especially under downward economic pressure [22]. Controlling the overinflated hospitalization rate has become a top priority for China’s healthcare delivery system. To be specific, effective reduction of avoidable hospitalization has been recognized as the way to control the overinflated hospitalization rate. In the above context, this study focused on analyzing the trend of avoidable hospitalization rate and the average per-case treatment cost of avoidable hospitalization in a large city in eastern China between 2015 and 2018 to provide evidence for Chinese policy makers and lay a solid foundation for other low- and middle-income countries (LMICs).

## Methods

The city investigated in this study is located in eastern China with a developed economy and a relatively well-developed health care service system. The vast majority of this city’s residents enjoy medical insurance in China, which consists of basic medical insurance for urban workers and basic medical insurance for urban and rural residents. Through the city’s health administration department, the hospital discharge data of all inpatients in the city from 2015 to 2018 were collected. The above data contain the key variables applied in this study, including gender, age, address, primary diagnosis, secondary diagnosis, length of stay, as well as hospitalization cost. No patients were involved in developing outcome measures and no analyses included any identifiable patient data.

Since hospitalization standards vary by country and health care system, there have been several international standards for avoidable hospitalization types, while there have been no similar standards in China. To ensure comparability with other nations, the standard proposed by the OECD Health Care Quality Indicators 2018–19 was used to determine avoidable hospitalization cases [7]. The indicator consists of six avoidable hospitalizations, including asthma, COPD, CHF, hypertension, diabetes, as well as diabetes-related lower limb amputation. Avoidable hospitalization cases were defined as inpatients being neither maternal nor neonatal, and the corresponding ICD-10 diagnosis codes were specified for different diseases [7]. For example, avoidable hospitalization cases for diabetes had a principal ICD-10 diagnosis code for a diabetes-related complication and the case for hypertension had a principal ICD-10 diagnosis code for essential hypertension or hypertension-related complications (for the list of diagnosis codes, see in the Additional file 1: Appendix 1). As diabetes-related lower limb amputation cases are rare and overlap with diabetes cases, five conditions (asthma, COPD, CHF, hypertension and diabetes) were selected for analysis. Besides, outpatient admissions, patients referred from another acute care facility, and any admissions with an ICD diagnosis code relating to pregnancy, labor and delivery, the puerperium, or the neonatal period or newborn were excluded. The avoidable hospitalization rate was obtained as “the number of avoidable hospitalizations in a given year/total hospitalizations in that year” [10, 14, 23].

From 2015 to 2018, a number of patients were hospitalized in the city from other regions, and the above hospitalizations may have an effect on the description of avoidable hospitalizations in the city. Accordingly, the data regarding hospitalization cases whose current address was not the address of the city was first excluded. We described the rate of avoidable hospitalizations, average cost and length of stay for avoidable hospitalization cases. Means and percentages were employed to describe continuous variables and categorical variables, respectively. The trends in the rate of avoidable hospitalizations were measured based on a linear probability model, and the trends in the average cost and length of stay for avoidable hospitalization cases were measured using a log-linear model. Specification of our model was as follows:

$${Y}_i={\beta}_0+{\beta}_1 Year+{\beta}_2 Age+{\beta}_3 Gender+{\beta}_4 CCI+{\beta}_5 Payment+{\updelta}_0$$

Where the dependent variable *Y*_*i*_ denotes whether it was an avoidable hospitalization case, the logarithm of the cost and length of stay for avoidable hospitalization. *Year* denotes the year of hospitalization. *Age, Gender, CCI and Payment* denotes patient characteristics, including age, gender, comorbidities as measured by the Charlson Comorbidity Index (CCI) [24] and the type of payment respectively. All expenditure variables are converted to 2015 using the Consumer Price Index (see in the Additional file 2: Appendix 2).

## Results

From 2015 to 2018, the absolute number of hospitalized cases in the city was 1,411,532, 1,725,357, 1,870,322 and 1,936,994, showing an increasing trend on a year-to-year basis; the absolute number of avoidable hospitalization cases was 81,095, 117,922, 106,087 and 125,372, thus indicating a trend of increasing while fluctuating (Table 1). In the four years, there were 6,944,205 hospitalizations in the city, of which 430,476 were avoidable hospitalizations, with an avoidable rate of nearly 6.20%. Avoidable hospitalizations occurred primarily in patients aged over 60 years (74.55%) and in male patients (54.10%), with a CCI of 0 exceeding 40%, followed by 1 and 2 points and above. Diabetes (32.72%) was the most common type of condition that occurred with avoidable hospitalization, followed by hypertension (28.92%) and chronic obstructive pulmonary disease (27.58%).

**Table 1:** Basic information regarding avoidable hospitalizations in 2015–18

Variables	2015	2016	2017	2018
N	81,095	117,922	106,087	125,372
Age				
15–40	3989	4519	5482	4722
	(4.92)	(3.83)	(5.17)	(3.77)
41–60	19,137	26,014	23,340	22,346
	(23.60)	(22.06)	(22.00)	(17.82)
61–80	38,627	56,981	49,331	43,339
	(47.63)	(48.32)	(46.50)	(34.57)
> 80	19,342	30,408	27,934	54,965
	(23.85)	(25.79)	(26.33)	(43.84)
Gender				
Female	36,951	55,034	48,400	57,209
	(45.57)	(46.67)	(45.62)	(45.63)
Male	44,144	62,888	57,687	68,163
	(54.43)	(53.33)	(54.38)	(54.37)
CCI				
0	33,766	49,786	42,759	48,405
	(41.64)	(42.22)	(40.31)	(38.61)
1	25,694	39,378	32,530	42,546
	(31.68)	(33.39)	(30.66)	(33.94)
≥2	21,635	28,758	30,798	34,421
	(26.68)	(24.39)	(29.03)	(27.46)
Conditions				
Hypertension	24,709	37,931	30,909	30,957
	(30.47)	(32.17)	(29.14)	(24.69)
Diabetes	25,743	35,163	37,979	41,975
	(31.74)	(29.82)	(35.80)	(33.48)
Asthma	2372	3410	2962	3060
	(2.92)	(2.89)	(2.79)	(2.44)
COPD	22,592	33,389	27,640	35,122
	(27.86)	(28.31)	(26.05)	(28.01)
CHF	5679	8029	6597	14,258
	(7.00)	(6.81)	(6.22)	(11.37)

From 2015 to 2018, the absolute number of avoidable hospitalizations for diabetes increased on a year-to-year basis, and the absolute number of avoidable hospitalizations for hypertension, asthma, chronic obstructive pulmonary disease, and congestive heart failure tended to increase while fluctuating, with the largest increase occurring for congestive heart failure. The overall avoidable hospitalization rate of the five conditions tended to increase while fluctuating, increasing from 5.75 to 6.83%, then decreasing to 5.67%, and subsequently increasing to 6.47%. The avoidable hospitalization rate of hypertension rose from 1.75 to 2.20% in 2016 before falling back to 1.60%, and the avoidable hospitalization rate of asthma increased from 0.17 to 0.20% in 2016 before falling back to 0.16% sequentially. In comparison, the avoidable hospitalization rate of diabetes increased from 1.82 to 2.04%, then decreased to 2.03%, then increased to 2.17%; the avoidable hospitalization rate of COPD increased from 1.60 to 1.94%, then decreased to 1.48%, then increased to 1.81%; and the avoidable hospitalization rate of CHF increased from 0.40 to 0.47%, then declined to 0.35% and then increased to 0.74% (Table 2).

**Table 2:** Change in the absolute number of avoidable hospitalizations and avoidable hospitalization rate, 2015–18

Year	Total	Hypertension	Diabetes	Asthma	COPD	CHF
2015	81,095(5.75)	24,709(1.75)	25,743(1.82)	2372(0.17)	22,592(1.60)	5679(0.40)
2016	117,922(6.83)[45.41]	37,931(2.20)[53.51]	35,163(2.04)[36.59]	3410(0.20)[43.76]	33,389(1.94)[47.79]	8029(0.47)[41.38]
2017	106,087(5.67)[−10.04]	30,909(1.65)[−18.51]	37,979(2.03)[8.01]	2962(0.16)[−13.14]	27,640(1.48)[−17.22]	6597(0.35)[−17.84]
2018	125,372(6.47)[18.18]	30,957(1.60)[0.16]	41,975(2.17)[10.52]	3060(0.16)[3.31]	35,122(1.81)[27.07]	14,258(0.74)[116.13]

Note: Avoidable hospitalization rate (%) is in small brackets, and the year-over-year growth rate (%) of avoidable hospitalizations is in middle brackets

For the average cost for avoidable hospitalization cases from 2015 to 2018, the average cost of avoidable hospitalizations declined from 11,152 yuan to 8644 yuan, from 11,678 yuan to 10,540 yuan, from 10,280 yuan to 8605 yuan, from 15,358 yuan to 13,685 yuan, and from 16,029 yuan to 13,787 yuan for hypertension, diabetes, asthma, COPD as well as CHF, respectively (Table 3).

**Table 3:** Change in average cost per avoidable hospitalization, 2015–18 (in Yuan)

Year	Hypertension	DM	Asthma	COPD	CHF
2015	11,152	11,678	10,280	15,358	16,029
2016	9802(−12.11)	11,257(−3.61)	9745(−5.20)	14,277(−7.04)	14,553(−9.21)
2017	9802(0.00)	11,471(1.90)	9337(−4.19)	14,610(2.33)	13,436(−7.68)
2018	8644(−11.81)	10,540(−8.12)	8605(− 7.84)	13,685(−6.33)	13,787(2.61)

Note: The year-over-year growth rate (%) of avoidable hospitalizations is in small brackets

For the average length of stay, the average length of stay for avoidable hospitalization cases for hypertension rose from 9.51 days to 10.00 days in 2016 from 2015 to 2018 and then fell to 6.82 days; that for diabetes rose from 10.50 days to 10.97 days in 2016 and then fell to 7.67 days; that for asthma rose from 8.61 days to 8.65 days and then fell back to 6.25 days; that for COPD declined from 11.08 days to 8.23 days on a year-to-year basis; that for CHF increased from 11.79 days to 15.19 days in 2017 and then decreased to 10.05 days in 2018 (Table 4).

**Table 4:** Change in average length of stay per avoidable hospitalization, 2015–18 (in days)

Year	Hypertension	DM	Asthma	COPD	CHF
2015	9.51	10.50	8.61	11.08	11.79
2016	10.00(5.15)	10.97(4.48)	8.65(0.46)	11.06(−0.18)	12.29(4.24)
2017	9.44(−5.60)	10.31(−6.02)	8.16(− 5.66)	10.98(− 0.72)	15.19(23.60)
2018	6.82(−27.75)	7.67(−25.61)	6.25(−23.41)	8.23(−25.05)	10.05(−33.84)

Note: The year-over-year growth rate (%) of avoidable hospitalizations is in small brackets

After the adjustment for linear probability models, the regression results (Table 5) indicated that the overall avoidable hospitalization rate of the five conditions from 2015 to 2018 significantly decreased by 0.16 percentage points (*P* < 0.01). The log-linear model regression results indicated that the average cost for avoidable hospitalization cases decreased by 14.24% (*P* < 0.01), and that the average length of stay for avoidable hospitalization cases from 2015 to 2018 decreased by 46.24% (*P* < 0.01).

**Table 5:** Regression results of avoidable hospitalizations

Variables	(1)	(2)	(3)
	avoidable hospitalization rate	hospitalization costs	length of stay
**Year (ref. 2015)**			
2016	0.0109***	− 0.0495***	0.0095***
	(0.000)	(0.003)	(0.003)
2017	−0.0010***	−0.0831***	− 0.0487***
	(0.000)	(0.004)	(0.003)
2018	−0.0016***	−0.1424***	− 0.4624***
	(0.000)	(0.003)	(0.003)
**Age group (ref. < 15)**			
15–40	0.0139***		
	(0.000)		
41–60	0.0480***	0.0486***	0.0147***
	(0.000)	(0.009)	(0.006)
61–80	0.0821***	0.1759***	0.1312***
	(0.000)	(0.009)	(0.006)
> 80	0.1036***	0.2893***	−0.0161***
	(0.000)	(0.009)	(0.006)
**Gender (ref. female)**			
Male	0.0155***	0.0829***	0.0413***
	(0.000)	(0.002)	(0.002)
**CCI group (ref. CCI = 0)**			
0 < CCI ≤ 1	0.0858***	0.1385***	0.1394***
	(0.000)	(0.003)	(0.003)
CCI ≥ 2	0.0390***	0.3333***	0.3440***
	(0.000)	(0.003)	(0.003)
**Payment method (ref. A)**			
B	0.0030***	−0.2680***	−0.1592***
	(0.000)	(0.003)	(0.003)
C	−0.0093***	−0.2042***	− 0.2279***
	(0.000)	(0.004)	(0.004)
D	−0.0136***	0.0694***	−0.5338***
	(0.000)	(0.005)	(0.004)
Constant	−0.0079***	8.8531***	2.0519***
	(0.000)	(0.009)	(0.006)
Observations	6,944,205	430,192	430,476
R-squared	0.058	0.086	0.196

Note: Each cell in columns 1–3 shows the coefficient from the regression; standard errors are reported in parentheses. The dependent variable is indicated in the column heading; the subsample is indicated in the row label. A = the Urban Employee Basic Medical Insurance, B = the Urban and Rural Residents Basic Medical Insurance, C = All self-pay, D = Other medical insurance. ***P < 0.01, **P < 0.05, *P < 0.1

## Discussion

This study aimed to describe trends in avoidable hospitalizations in a large, developed city in China, in which few similar studies have been conducted previously. As revealed by our findings, the number of avoidable hospitalization cases in the city increased yearly from 2015 to 2018, with the avoidable hospitalization rate fluctuating from 5.75 to 6.47%, while linear probability model regression results indicated a decrease of only 0.16 percentage points (*P* < 0.01). Avoidable hospitalizations occurred primarily in patients aged over 60 years (74.55%) and in male patients (54.10%). Diabetes (32.72%) was the most common type of disease among avoidable hospitalization cases, followed by hypertension (28.92%) and chronic obstructive pulmonary disease (27.58%). Compared with 2015, the average cost per hospitalization and average length of stay decreased for all five avoidable hospitalization types in 2018. For avoidable hospitalization rates, only the avoidable hospitalization rates for hypertension and asthma declined, while the avoidable hospitalization rates for the other three conditions rose.

In contrast, avoidable hospitalization rates of regions including Canada [25], Finland [26], Italy [14] and Singapore [10] have tended to decrease over the past few years. Numerous studies reported that continuity of care, a core element of primary care, is significantly correlated with reduced avoidable hospitalization rates [2]. An adequate supply of primary care physicians and a long-term relationship between primary care physicians and patients can decrease the incidence of avoidable hospitalizations [27]. Other studies also confirmed that maintaining an ongoing patient-physician relationship when treating chronic conditions will lead to higher satisfaction, better adherence, as well as fewer hospitalizations and emergency room visits [27, 28], thus indicating that increasing patients’ access to outpatient services may help reduce avoidable hospitalization rates.

From 2015 to 2018, the city introduced several policies to improve outpatient coverage for various chronic diseases (such as hypertension and diabetes) [29] and gradually introduced contracted family doctor services [28] to improve the accessibility and quality of primary health care services. However, the results of this study revealed that the effectiveness of the above measures has not yet been satisfactory. In particular, this study also reported a significant decreasing trend from 2015 to 2018 in the average cost (decreased by 14.24%) and the average length of stay (decreased by 46.24%) for avoidable hospitalization cases in the city, thus probably revealing that the number of avoidable hospitalization cases for minor illnesses may increase. On that basis, it was further indicated that the role of the primary care system and specialty clinics in the city is not improving, but rather tends to be functionally weakening.

This study found that avoidable hospitalizations occurred largely in patients aged over 60 years (74.55%) and in men (54.10%), consistent with the results of numerous similar studies [10, 30–32]. Older patients are a vulnerable group, which may suffer from multiple chronic diseases and underutilize primary health care services. We also found that more than 70% of avoidable hospitalization cases had a CCI less than or equal to 1, which could mean that strengthening primary health care services could have a significant impact on avoidable hospitalizations. As revealed by the above findings, policymakers may be required to focus more on providing primary health care services to vulnerable groups to reduce avoidable hospitalizations. We also found that the most common avoidable hospitalizations were for diabetes, hypertension and COPD, and that policymakers in the city could focus on the above diseases for chronic disease prevention and control.

There are still some limitations remaining to be addressed. We failed to obtain data on the underlying characteristics of population of the whole city, and therefore could not estimate the effect of population size on avoidable hospitalization rates. In addition, we did not get access to data for 2019–2021, so we were unable to describe the current trends of the city’s avoidable hospitalizations.

## Conclusion

According to the findings of this study, the avoidable hospitalization rate in this city in eastern China remains high, the absolute number of avoidable hospitalization cases is increasing on a year-to-year basis, and timely and effective primary health care services may help improve the above phenomena. This study has been one of the few to use hospital discharge data from a large Chinese city to describe avoidable hospitalizations, which can help gain insights into avoidable hospitalizations in China and provide a reference for similar countries. The occurrence of avoidable hospitalizations contributes to higher hospitalization rates and places a burden on health insurance funds. To reduce the incidence of avoidable hospitalizations, policymakers in the city should implement more effective policies to guide patients with avoidable hospitalizations, so as to more effectively exploit outpatient services and continuously improve the quality of primary care services.

## Supplementary Information

**Additional file 1: Appendix 1**

**Additional file 2: Appendix 2**

## Data Availability

The datasets generated and/or analyzed during the current study are not publicly available due to legal restrictions but are available from the corresponding author on reasonable request.

## Abbreviations

CCI: Charlson Comorbidity Index
CHF: Congestive heart failure
COPD: Chronic obstructive pulmonary disease
ICD: International Classification of Diseases
LMICs: Low- and middle-income countries
OECD: Organisation for Economic Co-operation and Development
