# Supplementary material for: Trends in avoidable hospitalizations in a developed City in eastern China: 2015 to 2018
Source: BMC Health Serv Res. 2022 Jul 4;22:856. doi: 10.1186/s12913-022-08275-w (PMC9252061; doi:10.1186/s12913-022-08275-w)
Supplement: Supplementary file 1 — Additional file 1: Appendix 1 [file 12913_2022_8275_MOESM1_ESM.pdf]

Appendix 1. Diagnosis code for an avoidable hospital admission for asthma, COPD, CHF, hypertension and diabetes (OECD Health Care Quality Indicators 2018-19)

Indicators in the Avoidable admission indicator set include:

1. Asthma hospital admission
2. Chronic obstructive pulmonary disease (COPD) hospital admission
3. Congestive heart failure (CHF) hospital admission
4. Hypertension hospital admission
5. Diabetes hospital admission

AA1) ASTHMA HOSPITAL ADMISSION

(See Glossary for definitions of italicized terminology)

Coverage: Population aged 15 and older (5 year age groups). All *acute care hospitals*, including public and private hospitals that provide inpatient care.

Numerator: All non-maternal/non-neonatal hospital *admissions* with a *principal diagnosis* code of asthma (see Asthma diagnosis codes below) in a specified year.

Exclude:

- Cases where the patient died in hospital during the admission.
- Cases resulting from a transfer from another acute care institution (*transfers-in*).
- Cases with *MDC 14* or specified pregnancy, childbirth, and puerperium codes in any field – Refer to Annex D (Excel sheet - HCQO 2018_19 Data Collection_Annex A-I)
- Cases with cystic fibrosis and anomalies of the respiratory system diagnosis code in any field (see ICD codes below)
- Cases that are *same day/day only admissions*

Denominator: Population count.

Asthma diagnosis codes:

ICD-9-CM	ICD-10-WHO
49300 EXTRINSIC ASTHMA NOS<br>49301 EXT ASTHMA W STATUS ASH<br>49302 EXT ASTHMA W ACUTE EXAC<br>49310 INT ASTHMA W/O STAT ASTH<br>49311 INTRINSIC ASTHMA NOS<br>49312 INT ASTHMA W ACUTE EXAC<br>49320 CH OB ASTH NOS<br>49321 CH OB ASTHMA W STAT ASTH<br>49322 CH OBS ASTH W ACUTE EXAC<br>49381 EXERCSE IND BRONCHOSPASM<br>49382 COUGH VARIANT ASTHMA<br>49390 ASTHMA NOS<br>49391 ASTHMA W STATUS ASTHMAT<br>49392 ASTHMA W ACUTE EXAC	J450 PREDOMINANTLY ALLERGIC ASTHMA<br>J451 NONALLERGIC ASTHMA<br>J458 MIXED ASTHMA<br>J459 ASTHMA, UNSPECIFIED<br>J46 STATUS ASTHMATICUS

Exclude diagnosis codes cystic fibrosis and anomalies of the respiratory system:

ICD-9-CM	ICD-10-WHO
27700 CYSTIC FIBROS W/O ILEUS	E840 CYSTIC FIBROSIS WITH PULMONARY MANIFESTATIONS
27701 CYSTIC FIBROS W ILEUS	E841 CYSTIC FIBROSIS WITH INTESTINAL MANIFESTATIONS
27702 CYSTIC FIBROS W PUL MAN	E848 CYSTIC FIBROSIS WITH OTHER MANIFESTATIONS
27703 CYSTIC FIBROSIS W GI MAN	E849 CYSTIC FIBROSIS, UNSPECIFIED
27709 CYSTIC FIBROSIS NEC	P27.0 WILSON-MIKITY SYNDROME
74721 ANOMALIES OF AORTIC ARCH	P27.1 BRONCHOPULMONARY DYSPLASIA ORIGINATING IN THE PERINATAL PERIOD
7483 LARYNGOTRACH ANOMALY NEC	P27.8 OTHER CHRONIC RESPIRATORY DISEASES ORIGINATING IN THE PERINATAL PERIOD
7484 CONGENITAL CYSTIC LUNG	P27.9 UNSPECIFIED CHRONIC RESP DISEASE ORIGINATING IN THE PERINATAL PERIOD
7485 AGENESIS OF LUNG	Q25.4 OTHER CONGENITAL MALFORMATIONS OF AORTA
74860 LUNG ANOMALY NOS	Q31.1 CONGENITAL SUBGLOTTIC STENOSIS
74861 CONGEN BRONCHIECTASIS	Q31.2 LARYNGEAL HYPOPLASIA
74869 LUNG ANOMALY NEC	Q31.3 LARYNGOCELE
7488 RESPIRATORY ANOMALY NEC	Q31.5 CONGENITAL LARYNGOMALACIA
7489 RESPIRATORY ANOMALY NOS	Q31.8 OTHER CONGENITAL MALFORMATIONS OF LARYNX
7503 CONG ESOPH FISTULA/ATRES	Q31.9 CONGENITAL MALFORMATION OF LARYNX, UNSPECIFIED
7593 SITUS INVERSUS	Q32.0 CONGENITAL TRACHEOMALACIA
7707 PERINATAL CHR RESP DIS	Q32.1 OTHER CONGENITAL MALFORMATIONS OF TRACHEA
	Q32.2 CONGENITAL BRONCHOMALACIA
	Q32.3 CONGENITAL STENOSIS OF BRONCHUS
	Q32.4 OTHER CONGENITAL MALFORMATIONS OF BRONCHUS
	Q33.0 CONGENITAL CYSTIC LUNG
	Q33.1 ACCESSORY LOBE OF LUNG
	Q33.2 SEQUESTRATION OF LUNG
	Q33.3 AGENESIS OF LUNG
	Q33.4 CONGENITAL BRONCHIECTASIS
	Q33.5 ECTOPIC TISSUE IN LUNG
	Q33.6 HYPOPLASIA AND DYSPLASIA OF LUNG
	Q33.8 OTHER CONGENITAL MALFORMATIONS OF LUNG
	Q33.9 CONGENITAL MALFORMATION OF LUNG, UNSPECIFIED
	Q34.0 ANOMALY OF PLEURA
	Q34.1 CONGENITAL CYST OF MEDIASTINUM
	Q34.8 OTHER SPECIFIED CONGENITAL MALFORMATIONS OF RESPIRATORY SYSTEM
	Q34.9 CONGENITAL MALFORMATION OF RESPIRATORY SYSTEM, UNSPECIFIED
	Q39.0 ATRESIA OF OESOPHAGUS WITHOUT FISTULA
	Q39.1 ATRESIA OF OESOPHAGUS WITH TRACHEO-OESOPHAGEAL FISTULA
	Q39.2 CONGENITAL TRACHEO-OESOPHAGEAL FISTULA WITHOUT ATRESIA
	Q39.3 CONGENITAL STENOSIS AND STRICTURE OF OESOPHAGUS
	Q39.4 OESOPHAGEAL WEB
	Q39.8 OTHER CONGENITAL MALFORMATIONS OF OESOPHAGUS
	Q89.3 SITUS INVERSUS

AA2) CHRONIC OBSTRUCTIVE PULMONARY DISEASE (COPD) HOSPITAL ADMISSION

(See Glossary for definitions of italicized terminology)

Coverage: Population aged 15 and older (5 year age groups). All *acute care hospitals*, including public and private hospitals that provide inpatient care.

Numerator: All non-maternal/non-neonatal hospital *admissions* with a *principal diagnosis* code of Chronic Obstructive Pulmonary Disease (See COPD diagnosis codes below) in a specified year.

Exclude:

- Cases where the patient died in hospital during the admission.
- Cases resulting from a transfer from another acute care institution (*transfers-in*).
- Cases with *MDC 14* or specified pregnancy, childbirth, and puerperium codes in any field – Refer to Annex D (Excel sheet - HCQO 2018_19 Data Collection_Annex A-I)
- Cases that are *same day/day only admissions*

Denominator: Population count.

COPD diagnosis codes:

ICD-9-CM	ICD-10-WHO
490 BRONCHITIS NOS*<br>4660 AC BRONCHITIS*<br>4910 SIMPLE CHR BRONCHITIS<br>4911 MUCOPURUL CHR BRONCHITIS<br>49120 OBS CHR BRNC W/O ACT EXA<br>49121 OBS CHR BRNC W ACT EXA<br>4918 CHRONIC BRONCHITIS NEC<br>4919 CHRONIC BRONCHITIS NOS<br>4920 EMPHYSEMATOUS BLEB<br>4928 EMPHYSEMA NEC<br>494 BRONCHIECTASIS<br>4940 BRONCHIECTAS W/O AC EXAC<br>4941 BRONCHIECTASIS W AC EXAC<br>496 CHR AIRWAY OBSTRUCT NEC<br><br>* Qualifies only if accompanied by secondary diagnosis of 491.xx, 492.x, 494.x or 496 (i.e., any other code on this list).	J40 BRONCHITIS*<br>J410 SIMPLE CHRONIC BRONCHITIS<br>J411 MUCOPURULENT CHRONIC BRONCHITIS<br>J418 MIXED SIMPLE AND MUCOPURULENT CHRONIC BRONCHITIS<br>J42 UNSPECIFIED CHRONIC BRONCHITIS<br>J430 MACLEOD'S SYNDROME<br>J431 PANLOBULAR EMPHYSEMA<br>J432 CENTRILOBULAR EMPHYSEMA<br>J438 OTHER EMPHYSEMA<br>J439 EMPHYSEMA, UNSPECIFIED<br>J440 COPD WITH ACUTE LOWER RESPIRATORY INFECTION<br>J441 COPD WITH ACUTE EXACERBATION, UNSPECIFIED<br>J448 OTHER SPECIFIED CHRONIC OBSTRUCTIVE PULMONARY DISEASE<br>J449 CHRONIC OBSTRUCTIVE PULMONARY DISEASE, UNSPECIFIED<br>J47 BRONCHIECTASIS<br><br>* Qualifies only if accompanied by secondary diagnosis of J41, J43, J44, J47

AA3) CONGESTIVE HEART FAILURE (CHF) HOSPITAL ADMISSION

(See Glossary for definitions of italicized terminology)

Coverage: Population aged 15 and older (5 year age groups). All *acute care hospitals*, including public and private hospitals that provide inpatient care.

Numerator: All non-maternal/non-neonatal hospital *admissions* with *principal diagnosis* code of Congestive Heart Failure (See CHF diagnosis codes below) in a specified year.

Exclude:

- Cases where the patient died in hospital during the admission.
- Cases resulting from a transfer from another acute care institution (*transfers-in*).
- Cases with cardiac procedure codes in any field – Refer to Annex A (Excel sheet - HCQO 2018_19 Data Collection_Annex A-I)
- Cases with *MDC 14* or specified pregnancy, childbirth, and puerperium codes in any field – Refer to Annex D (Excel sheet - HCQO 2018_19 Data Collection_Annex A-I)
- Cases that are *same day/day only admissions*

Denominator: Population count.

CHF diagnosis codes:

ICD-9-CM	ICD-10-WHO
39891 RHEUMATIC HEART FAILURE<br>40201 MAL HYPERT HRT DIS W CHF<br>40211 BENIGN HYP HRT DIS W CHF<br>40291 HYPERTEN HEART DIS W CHF<br>40401 MAL HYPER HRT/REN W CHF<br>40403 MAL HYP HRT/REN W CHF/RF<br>40411 BEN HYPER HRT/REN W CHF<br>40413 BEN HYP HRT/REN W CHF/RF<br>40491 HYPER HRT/REN NOS W CHF<br>40493 HYP HT/REN NOS W CHF/RF<br>4280 CONGESTIVE HEART FAILURE<br>4281 LEFT HEART FAILURE<br>42820 SYSTOLIC HRT FAILURE NOS<br>42821 AC SYSTOLIC HRT FAILURE<br>42822 CHR SYSTOLIC HRT FAILURE<br>42823 AC ON CHR SYST HRT FAIL<br>42830 DIASTOLC HRT FAILURE NOS<br>42831 AC DIASTOLIC HRT FAILURE<br>42832 CHR DIASTOLIC HRT FAIL	I11.0 HYPERTENSIVE HEART DISEASE WITH (CONGESTIVE) HEART FAILURE<br>I13.0 HYPERTENSIVE HEART AND RENAL DISEASE WITH (CONGESTIVE) HEART FAILURE<br>I13.2 HYPERTENSIVE HEART AND RENAL DISEASE WITH BOTH (CONGESTIVE) HEART FAILURE AND RENAL FAILURE<br>I50.0 CONGESTIVE HEART FAILURE<br>I50.1 LEFT VENTRICULAR FAILURE<br>I50.9 HEART FAILURE, UNSPECIFIED

42833 AC ON CHR DIAST HRT FAIL<br>42840 SYST/DIAST HRT FAIL NOS<br>42841 AC SYST/DIASTOL HRT FAIL<br>42842 CHR SYST/DIASTL HRT FAIL<br>42843 AC/CHR SYST/DIA HRT FAIL<br>4289 HEART FAILURE NOS	

AA4) HYPERTENSION HOSPITAL ADMISSION

(See Glossary for definitions of italicized terminology)

Coverage: Population aged 15 and older (5 year age groups). All *acute care hospitals*, including public and private hospitals that provide inpatient care.

Numerator: All non-maternal/non-neonatal hospital *admissions* with *principal diagnosis* code of Hypertension (see Hypertension diagnosis codes below) in a specified year.

Exclude:

- Cases where the patient died in hospital during the admission.
- Cases resulting from a transfer from another acute care institution (*transfers-in*).
- Cases with cardiac procedure codes in any field – Refer to Annex A (Excel sheet - HCQO 2018_19 Data Collection_Annex A-I)
- Cases with *MDC 14* or specified pregnancy, childbirth, and puerperium codes in any field – Refer to Annex D (Excel sheet - HCQO 2018_19 Data Collection_Annex A-I)
- Cases that are *same day/day only admissions*

Denominator: Population count.

Hypertension diagnosis codes:

ICD-9-CM	ICD-10-WHO
4010 MALIGNANT HYPERTENSION<br>4019 HYPERTENSION NOS<br>40200 MAL HYPERTEN HRT DIS NOS<br>40210 BEN HYPERTEN HRT DIS NOS<br>40290 HYPERTENSIVE HRT DIS NOS<br>40300 MAL HYP REN W/O REN FAIL<br>40310 BEN HYP REN W/O REN FAIL<br>40390 HYP REN NOS W/O REN FAIL<br>40400 MAL HY HT/REN W/O CHF/RF<br>40410 BEN HY HT/REN W/O CHF/RF<br>40490 HY HT/REN NOS W/O CHF/RF	I10 ESSENTIAL (PRIMARY) HYPERTENSION<br>I119 HYPERTENSIVE HEART DISEASE WITHOUT (CONGESTIVE) HEART FAILURE<br>I129 HYPERTENSIVE RENAL DISEASE WITHOUT RENAL FAILURE<br>I139 HYPERTENSIVE HEART AND RENAL DISEASE, UNSPECIFIED

AA5) DIABETES HOSPITAL ADMISSION

(See Glossary for definitions of italicized terminology)

Coverage: Population aged 15 and older (5 year age groups). All *acute care hospitals*, including public and private hospitals that provide inpatient care.

Numerator: All non-maternal/non-neonatal hospital *admissions* with a *principal diagnosis* code of diabetes (see Diabetes diagnosis codes below) in a specified year.

Exclude:

- Cases where the patient died in hospital during the admission.
- Cases resulting from a transfer from another acute care institution (*transfers-in*).
- Cases with *MDC 14* or specified pregnancy, childbirth, and puerperium codes in any field – Refer to Annex D (Excel sheet - HCQO 2018_19 Data Collection_Annex A-I)
- Cases that are *same day/day only admissions*

Denominator: Population count.

Diabetes diagnosis codes

ICD-9-CM	ICD-10-WHO
25002 DMII WO CMP UNCNTRLD<br>25003 DMI WO CMP UNCNTRLD<br>25010 DMII KETO NT ST UNCNTRLD<br>25011 DMI KETO NT ST UNCNTRLD<br>25012 DMII KETOACD UNCONTROLD<br>25013 DMI KETOACD UNCONTROLD<br>25020 DMII HPRSM NT ST UNCNTRL<br>25021 DMI HPRSM NT ST UNCNTRLD<br>25022 DMII HPROSMLR UNCONTROLD<br>25023 DMI HPROSMLR UNCONTROLD<br>25030 DMII O CM NT ST UNCNTRLD<br>25031 DMI O CM NT ST UNCNTRL<br>25032 DMII OTH COMA UNCONTROLD<br>25033 DMI OTH COMA UNCONTROLD<br>25040 DMII RENL NT ST UNCNTRLD<br>25041 DMI RENL NT ST UNCNTRLD<br>25042 DMII RENAL UNCNTRLD<br>25043 DMI RENAL UNCNTRLD<br>25050 DMII OPHTH NT ST UNCNTRL<br>25051 DMI OPHTH NT ST UNCNTRLD<br>25052 DMII OPHTH UNCNTRLD<br>25053 DMI OPHTH UNCNTRLD<br>25060 DMII NEURO NT ST UNCNTRL<br>25061 DMI NEURO NT ST UNCNTRLD	E10.0 INSULIN-DEPENDENT DIABETES MELLITUS WITH COMA<br>E10.1 INSULIN-DEPENDENT DIABETES MELLITUS WITH KETOACIDOSIS<br>E10.2 INSULIN-DEPENDENT DIABETES MELLITUS WITH RENAL COMPLICATIONS<br>E10.3 INSULIN-DEPENDENT DIABETES MELLITUS WITH OPHTHALMIC COMPLICATIONS<br>E10.4 INSULIN-DEPENDENT DIABETES MELLITUS WITH NEUROLOGICAL COMPLICATIONS<br>E10.5 INSULIN-DEPENDENT DM WITH PERIPHERAL CIRCULATORY COMPLICATIONS<br>E10.6 INSULIN-DEPENDENT DM WITH OTHER SPECIFIED COMPLICATIONS<br>E10.7 INSULIN-DEPENDENT DIABETES MELLITUS WITH MULTIPLE COMPLICATIONS<br>E10.8 INSULIN-DEPENDENT DIABETES MELLITUS WITH UNSPECIFIED COMPLICATIONS<br>E10.9 INSULIN-DEPENDENT DIABETES MELLITUS WITHOUT COMPLICATIONS<br>E11.0 NON-INSULIN-DEPENDENT DIABETES MELLITUS WITH COMA<br>E11.1 NON-INSULIN-DEPENDENT DIABETES MELLITUS WITH KETOACIDOSIS

25062 DMII NEURO UNCNTRLD<br>25063 DMI NEURO UNCNTRLD<br>25070 DMII CIRC NT ST UNCNTRLD<br>25071 DMI CIRC NT ST UNCNTRLD<br>25072 DMII CIRC UNCNTRLD<br>25073 DMI CIRC UNCNTRLD<br>25080 DMII OTH NT ST UNCNTRLD<br>25081 DMI OTH NT ST UNCNTRLD<br>25082 DMII OTH UNCNTRLD<br>25083 DMI OTH UNCNTRLD<br>25090 DMII UNSPF NT ST UNCNTRL<br>25091 DMI UNSPF NT ST UNCNTRLD<br>25092 DMII UNSPF UNCNTRLD<br>25093 DMI UNSPF UNCNTRLD	E11.2 NON-INSULIN-DEPENDENT DIABETES MELLITUS WITH RENAL COMPLICATIONS<br>E11.3 NON-INSULIN-DEPENDENT DMWITH OPHTHALMIC COMPLICATIONS<br>E11.4 NON-INSULIN-DEPENDENT DM WITH NEUROLOGICAL COMPLICATIONS<br>E11.5 NON-INSULIN-DEPENDENT DM WITH PERIPHERAL CIRCULATORY COMPLICATIONS<br>E11.6 NON-INSULIN-DEPENDENT DM WITH OTHER SPECIFIED COMPLICATIONS<br>E11.7 NON-INSULIN-DEPENDENT DIABETES MELLITUS WITH MULTIPLE COMPLICATIONS<br>E11.8 NON-INSULIN-DEPENDENT DM WITH UNSPECIFIED COMPLICATIONS<br>E11.9 NON-INSULIN-DEPENDENT DIABETES MELLITUS WITHOUT COMPLICATIONS<br>E13.0 OTHER SPECIFIED DIABETES MELLITUS WITH COMA<br>E13.1 OTHER SPECIFIED DIABETES MELLITUS WITH KETOACIDOSIS<br>E13.2 OTHER SPECIFIED DIABETES MELLITUS WITH RENAL COMPLICATIONS<br>E13.3 OTHER SPECIFIED DIABETES MELLITUS WITH OPHTHALMIC COMPLICATIONS<br>E13.4 OTHER SPECIFIED DIABETES MELLITUS WITH NEUROLOGICAL COMPLICATIONS<br>E13.5 OTHER SPECIFIED DM WITH PERIPHERAL CIRCULATORY COMPLICATIONS<br>E13.6 OTHER SPECIFIED DIABETES MELLITUS WITH OTHER SPECIFIED COMPLICATIONS<br>E13.7 OTHER SPECIFIED DIABETES MELLITUS WITH MULTIPLE COMPLICATIONS<br>E13.8 OTHER SPECIFIED DIABETES MELLITUS WITH UNSPECIFIED COMPLICATIONS<br>E13.9 OTHER SPECIFIED DIABETES MELLITUS WITHOUT COMPLICATIONS<br>E14.0 UNSPECIFIED DIABETES MELLITUS WITH COMA<br>E14.1 UNSPECIFIED DIABETES MELLITUS WITH KETOACIDOSIS<br>E14.2 UNSPECIFIED DIABETES MELLITUS WITH RENAL COMPLICATIONS<br>E14.3 UNSPECIFIED DIABETES MELLITUS WITH OPHTHALMIC COMPLICATIONS<br>E14.4 UNSPECIFIED DIABETES MELLITUS WITH NEUROLOGICAL COMPLICATIONS<br>E14.5 UNSPECIFIED DM WITH PERIPHERAL CIRCULATORY COMPLICATIONS<br>E14.6 UNSPECIFIED DIABETES MELLITUS WITH OTHER SPECIFIED COMPLICATIONS<br>E14.7 UNSPECIFIED DIABETES MELLITUS WITH MULTIPLE COMPLICATIONS<br>E14.8 UNSPECIFIED DIABETES MELLITUS WITH UNSPECIFIED COMPLICATIONS<br>E14.9 UNSPECIFIED DIABETES MELLITUS WITHOUT COMPLICATIONS
