# Supplementary material for: Trends in avoidable hospitalizations in a developed City in eastern China: 2015 to 2018
Source: BMC Health Serv Res. 2022 Jul 4;22:856. doi: 10.1186/s12913-022-08275-w (PMC9252061; doi:10.1186/s12913-022-08275-w)
Supplement: Supplementary file 2 — Additional file 2: Appendix 2 [file 12913_2022_8275_MOESM2_ESM.docx]

Table 6. CPI adjusted

| year | unadjusted | adjusted（2015 baseline） | CPI%, The year-earlier index was 100 |
| --- | --- | --- | --- |
| 2015 | Y1 | Y1 | 100 |
| 2016 | Y2 | Y2/102.7% | 102.7 |
| 2017 | Y3 | Y3/102.7%/102.3% | 102.3 |
| 2018 | Y4 | Y4/102.7%/102.3%/102.4% | 102.4 |

All expenditure variables are converted to 2015 using the Consumer Price Index.
